# The contribution of ultrasound in the diagnostic pathway of a symptomatic hepatocellular adenoma arising from ectopic liver

**DOI:** 10.1007/s40477-024-00872-1

**Published:** 2024-02-23

**Authors:** Nicholas Vanigli, Laura Melotti, Nikolas Dussias, Amalia Sanna Passino, Elena Mazzotta, Chiara De Molo, Lorenzo Gentilini, Gilberto Poggioli, Paolo Gionchetti, Fernando Rizzello

**Affiliations:** University Hospital of Bologna Sant’Orsola-Malpighi Polyclinic, IRCCS Azienda Ospedaliero, Universitaria Di Bologna Policlinico Di Sant’Orsola, Bologna, Italy

**Keywords:** Hepatocellular adenoma, Ectopic liver, Ultrasound

## Abstract

Ectopic liver (EL) is a rare congenital anomaly characterized by the presence of a mass composed of hepatic tissue localized in a different anatomical location with no connection to the native liver. Usually an incidental finding, EL can rarely cause symptoms such as abdominal pain due to torsion, intraperitoneal bleeding, compression, obstruction, or neoplastic transformation, both benign and malignant. EL is often suspected after instrumental investigations such as ultrasound, CT and MRI, however a definitive diagnosis is necessarily bioptic. Here we report a case of a 22-year-old Italian female patient with acute abdominal pain, who underwent abdominal ultrasound, CEUS with Sonovue®, CT scan and ultrasound-guided biopsy which raised the suspicion of hepatocellular adenoma (H-HCA). After a laparoscopic excision of the lesion a diagnosis of H-HCA was formulated.

## Introduction

Ectopic liver (EL) is a rare congenital anomaly characterized by the presence of a mass composed of hepatic tissue localized in a different anatomical location with no connection to the native liver. Incidence varies between 0.47% and 0.7%[1]. The mass can be mainly found in abdominal organs including gallbladder, spleen, jejunum, stomach, retroperitoneum, pancreas, adrenal gland, diaphragm, inferior vena cava, and the heart[2]. EL is usually an incidental finding, that can rarely cause symptoms such as abdominal pain due to torsion, intraperitoneal bleeding, compression, obstruction, or neoplastic transformation. EL is often suspected after instrumental investigations such as ultrasound, CT and MRI, however a definitive diagnosis is necessarily bioptic.

In this article, we present a case of abdominal pain caused by a hepatocellular adenoma (H-HCA) arising from EL.

## Case description

A 22-year-old female patient with no relevant clinical history presented to our clinic with acute abdominal pain. Physical examination revealed a tender, palpable mass in the lower right quadrant. Laboratory tests showed mild signs of systemic inflammation and liver enzyme alteration (WBC 13.36 × 10^9/L; CRP 4.89 mg/dL; AST 141 IU/L; and ALT 110 IU/L).

**Fig. 1:**
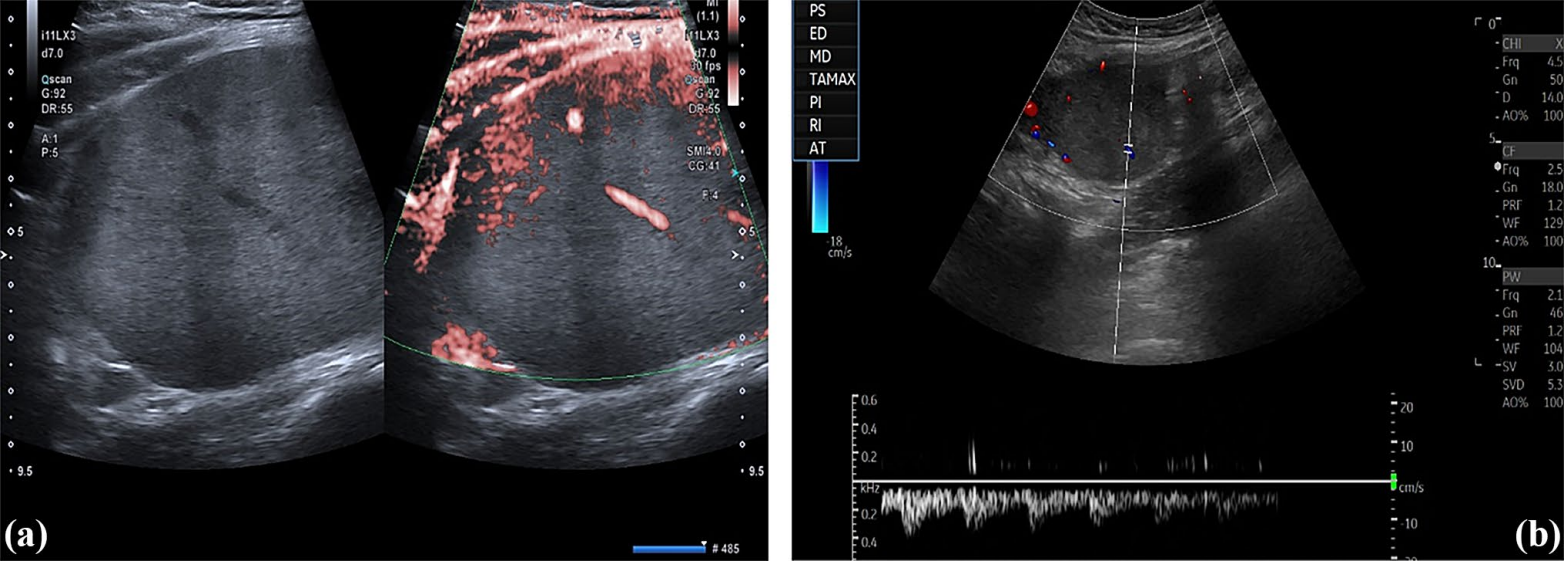
Echoic mass in right lower quadrant with some intralesional vascular signals (**a**). Color-Doppler detected low-resistance arterial vascular signals (**b**)

**Fig. 2:**
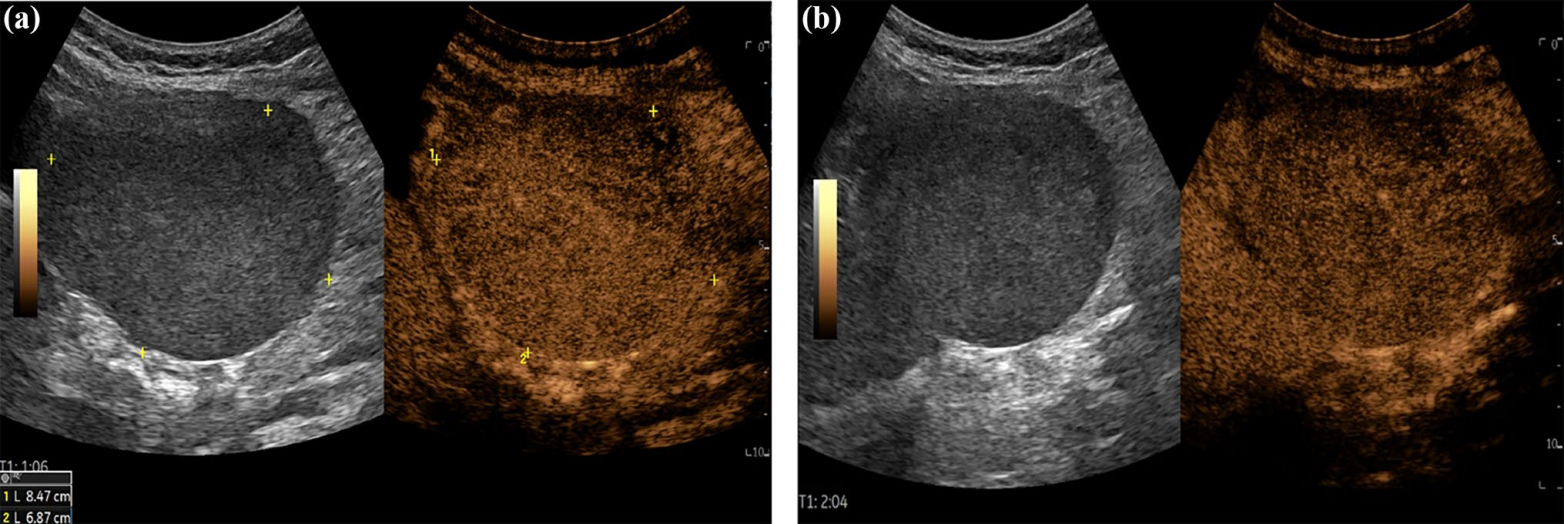
CEUS images at 1 min (**a**) and at 2 min (**b**) from the Sonovue® injection. CEUS showed arterial phase enhancement with weak and late washout

Abdominal ultrasound was performed showing a bulky, solid, round, echoic formation with well-defined margins in the right lower abdominal quadrant, with an inhomogeneous echostructure and low-resistance arterial Color-Doppler vascular signals (Fig. 1). This mass was cleaved from all the adjacent structures—including the abdominal muscles. So, it was considered appropriate proceeding using CEUS with Sonovue® (Bracco, Milano, Italy). Sonovue® was injected into the antecubital vein in a bolus fashion of 2.5 mL, followed by a flush of 10 mL of 0.9% normal saline solution. Continuous scanning began immediately and lasted 4–5 min following contrast injection. CEUS showed arterial phase enhancement with weak and late washout (Fig. 2).

The gynecologic origin of the lesion was excluded after a transvaginal ultrasound was performed.

**Fig. 3:**
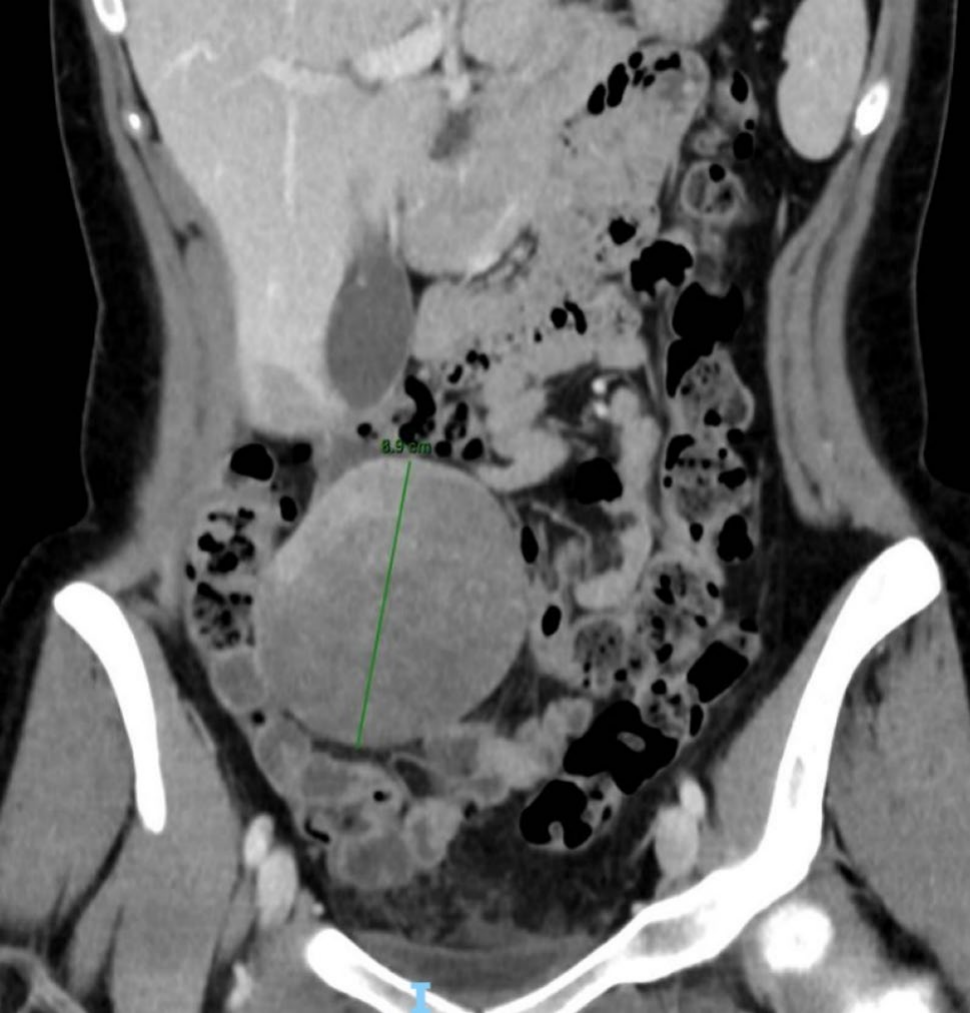
Abdominal CT scan with coronal reconstruction showed a solid formation with well-defined margins, measuring 86 × 65 × 88 mm, with progressive, not homogeneous late contrast enhancement, especially at the superior pole

Abdominal CT confirmed the presence of a solid formation in the lower right quadrant measuring 86 × 65x88 mm, with progressive, not homogeneous late contrast enhancement, especially at the superior pole (Fig. 3). The patient underwent an ultrasound-guided biopsy which raised the suspicion of H-HCA.

Finally, the patient was admitted for surgery, laparoscopic excision of the lesion was performed. Histological examination of the mass revealed a benign hepatocellular tumor with well-defined margins, macro- and mediovesicular steatosis, ballooning degeneration and cytoplasmic clearing, without cytological atypia. A diagnosis of ectopic liver with HNF-1α-mutated H-HCA was made.

## Discussion

Here we discuss an extremely rare case of HCA in symptomatic EL, diagnosed by abdominal ultrasound, contrast-enhanced CT and ultrasound-guided biopsy. Generally, these findings are incidental as they are often asymptomatic, therefore EL is usually found during surgery or as an autoptic finding.

The ectopic HCA described in this case presented common aspects to the classical liver adenomas at US and CEUS, in accordance with an Italian multicenter experience[3]. In fact, Color-Doppler detected intralesional venous vascular signals and low-resistance perilesional arterial vascular signals. CEUS showed a homogeneous arterial phase enhancement with a centripetal filling pattern, which is opposite to the pattern of centrifugal filling, typical of focal nodular hyperplasia (FNH)[3, 4]. Moreover, CEUS also showed a weak and late washout, with more hypoenhancement in the late phase compared to FNH[4]. Despite this, compared to classical HCA, in our case B mode US showed more clearly inhomogeneous echostructure with necrotic-hemorrhagic areas probably due to the lesion relevant size.

Cases of EL are very rare, the first being reported in 1922[5]. Many theories have been presented to explain the development of EL. In particular, the liver is formed from a rudimentary budding of the ventral endoderm of the midgut. The hepatic diverticulum is differentiated into the cranial portion (pars hepatica—future liver) and caudal portion (pars cystica—future gallbladder and cystic duct). Pars hepatica emits proliferations of solid hepatic cords in the mesenchyme adjacent to the transverse septum, forming blood sinusoids surrounded by liver cells. Ingrowth of the bile canaliculi (from the pars cystica) to form the portal triad occurs after the proliferation of the hepatic cords. Therefore, ectopia can be a consequence of early or late developmental abnormalities of liver tissue as it migrates aberrantly into the transverse septum or onto the pars cystica[6].

In EL, both benign and malignant lesions can develop. Benign lesions are less frequent and only four cases of extracapsular HCA have been described: a 50-year-old woman with biochemical cholestasis without symptoms[7]; a 43-year-old woman in therapy with oral contraceptives presented to the emergency department with left shoulder pain[8]; a 35-year-old woman with abdominal pain and a palpable mass in the right hypochondrium [9]; a 60-year-old woman admitted to the general surgery department with nonspecific abdominal pain[10].

Malignant tumors are common because EL is more prone to neoplastic degeneration than normal hepatic tissue. Indeed, it is metabolically disadvantaged as it lacks proper vascularization, which can facilitate carcinogenesis[1].

Upon the risk of carcinogenesis, surgical resection is recommended even for incidental EL. Moreover, in our patient laparoscopic surgical excision was performed since there was a high risk of rupture and bleeding.

## Conclusion

To the best of our knowledge this is the fifth report in literature of HCA arising from EL and the third case revealed by abdominal ultrasound. In this case, the patient presented with abdominal pain in the right lower quadrant and the differential diagnosis included acute appendicitis, ovarian torsion and terminal ileitis; however abdominal and transvaginal ultrasound were fundamental in the work-up, allowing for quick referral to radiology and subsequently surgery.

## Data Availability

All data underlying the results are available as part of the article and no additional source data are required.
